# Differential P300 Signatures of Executive Dysfunction in Attention-Deficit/Hyperactivity Disorder and Borderline Intellectual Functioning

**DOI:** 10.5152/eurasianjmed.2026.261539

**Published:** 2026-06-30

**Authors:** Elif Abanoz, Onur Erdem Korkmaz, Adil Deniz Duru, İbrahim Selçuk Esin

**Affiliations:** 1 Department of Child and Adolescent Psychiatry, Sivas Cumhuriyet University Faculty of Medicine, Sivas, Türkiye; 2 Department of Electrical and Electronic Engineering, Atatürk University Faculty of Engineering, Erzurum, Türkiye; 3 Sports Coaching Education, Marmara University Faculty of Sport Sciences, İstanbul, Türkiye; 4 Department of Child and Adolescent Psychiatry, Kanuni Training and Research Hospital, Trabzon University Faculty of Medicine, Trabzon, Türkiye

**Keywords:** ADHD, borderline intellectual functioning, child psychiatry, event-related potentials, executive function, P300

## Abstract

**Background:**

Attention-deficit/hyperactivity disorder (ADHD) and borderline intellectual functioning (BIF) are developmental conditions frequently characterized by executive dysfunction. However, the cognitive and neurophysiological differences between these conditions remain insufficiently defined. This study aimed to examine the behavioral and electrophysiological features of executive function in children with ADHD and BIF.

**Methods:**

Children with ADHD and BIF, along with typically developing controls, completed computerized Stroop and Go/No-Go tasks while electroencephalography was recorded. Behavioral performance was evaluated using reaction times, accuracy indices, omission errors, and commission errors. Electrophysiological analyses focused on P300 event-related potential amplitude and latency at frontal (F3, F4) and central (C3) electrode sites.

**Results:**

Children with BIF demonstrated slower reaction times, increased omission errors, and prolonged P300 latency at the F4 electrode, indicating reduced processing speed and attentional inefficiency. In contrast, children with ADHD exhibited faster but more error-prone response patterns, characterized by increased commission errors and shorter P300 latency, consistent with impulsivity and impaired inhibitory control.

**Conclusion:**

Attention-deficit/hyperactivity disorder and BIF share executive dysfunction but differ in underlying cognitive and neurophysiological profiles. Attention-deficit/hyperactivity disorder is primarily associated with disinhibition, whereas BIF is characterized by reduced processing speed and impaired sustained attention. Differences in P300 patterns may help distinguish between these conditions and support diagnosis and individualized intervention strategies.

**Main Points**

- Attention-deficit/hyperactivity disorder (ADHD) and borderline intellectual functioning (BIF) show distinct behavioral profiles of executive dysfunction in pediatric populations.
- Children with BIF exhibit slower reaction times, increased omission errors, and prolonged P300 latency at F4, reflecting cognitive slowing and attentional lapses.
- Children with ADHD demonstrate faster responses, higher commission errors, and shorter P300 latency, indicative of impulsivity and reduced inhibitory control.
- P300 amplitude and latency analyses at F3, F4, and C3 reveal differential engagement of frontal and motor control mechanisms across ADHD and BIF.
- Event-related potential measures provide convergent neurophysiological markers that may inform diagnosis and guide targeted interventions for executive dysfunction in ADHD and BIF.

## Introduction

Attention deficit hyperactivity disorder (ADHD) is a common neurodevelopmental disorder defined by inattention, hyperactivity, and impulsivity, with marked impairments in executive control that persist across the lifespan.^1^ These impairments primarily involve executive functions such as attention, inhibitory control, working memory, and monitoring of performance. Meta-analyses consistently demonstrate that executive dysfunction is a core neurocognitive feature of ADHD.^2^

Borderline intellectual functioning (BIF), defined as intelligence quotient (IQ) scores between 70 and 85, represents an intermediate range between intellectual disability and average intelligence.^3^ Individuals with BIF frequently show deficits in memory, reasoning, and inhibitory control, which manifest in adaptive, social, and daily living difficulties.^3^^,^^4^ Despite overlapping executive dysfunction, direct comparisons of ADHD and BIF are scarce.^3^

Event-related potentials (ERPs) provide a noninvasive measure of neural activity linked to sensory, cognitive, and motor processes during information processing.^5^ Among ERP components, the P300 is widely studied as an index of attention, response inhibition, and working memory, with amplitude and latency reflecting the efficiency of cognitive processing.^6^^,^^7^ Altered P300 activity has been reported across psychiatric disorders, including ADHD, depression, and schizophrenia. Event-related potential studies in ADHD consistently demonstrate reduced P300 amplitude, reflecting deficits in attentional filtering, stimulus evaluation, and inhibitory control, although findings regarding P300 latency remain inconsistent.^8^^,^^9^ In contrast, the limited ERP literature in individuals with borderline intellectual functioning suggests prolonged P200, N200, and P300 latencies without consistent alterations in amplitude.^10^

The present study aims to investigate behavioral and electrophysiological correlates of executive function in children with ADHD and BIF compared to typically developing controls. Using computerized Stroop and Go/No-Go tasks alongside electroencephalography recordings, the reaction times, accuracy, omission and commission errors, and P300 amplitude and latency at frontal and central electrode sites were examined. It was hypothesized that children with ADHD would exhibit faster responses, increased commission errors, and altered P300 profiles consistent with impulsivity and reduced inhibitory control, whereas children with BIF would show slower reaction times, higher omission errors, and prolonged P300 latency, reflecting attentional difficulties and cognitive slowing. This study aims to delineate distinct cognitive and neural profiles of executive functioning in ADHD and BIF, enhancing understanding of shared and divergent mechanisms underlying executive dysfunction in childhood.

## Material and Methods

### Participants

Ethical approval for the study was obtained from the Ethics Committee of Atatürk University Faculty of Medicine (Approval No. 11; Date: November 7, 2019). The study was funded by the Scientific Research Projects Coordination Unit under project code TTU-2020-8327. The study included 3 groups of right-hand–dominant participants aged 10-16 years, matched for age, gender, and socioeconomic status. The ADHD group consisted of 23 participants diagnosed with ADHD, the BIF group included 21 participants diagnosed with BIF, and the Control group comprised 22 healthy participants. Participants in the ADHD and BIF groups were selected from patients who presented to the Child and Adolescent Psychiatry Outpatient Clinic, whereas participants in the Control group were randomly selected from healthy children and adolescents admitted to the Pediatrics Clinic. The study was conducted between July 2020 and August 2020. The shared exclusion criteria for the ADHD and BIF groups encompassed a diagnosis of neurodevelopmental disorders (e.g., autism spectrum disorder, learning disability, intellectual disability), sensory impairments (hearing or visual), current psychiatric disorders (e.g., mania, psychosis), chronic medical conditions (e.g., neurological or endocrine diseases), regular medication use, a history of head trauma, and substance use (e.g., tobacco, alcohol, or illicit drugs). For the ADHD group, an additional exclusion criterion was a diagnosis of BIF, whereas for the BIF group, an additional exclusion criterion was a diagnosis of ADHD.

### Procedure

Participants and their parents were provided with comprehensive information regarding the study, and written informed consent was obtained from the parents. To evaluate the participants’ mental health status, parents were interviewed by a clinician using the Development and Well-Being Assessment (DAWBA) via computer applications. The DAWBA is a structured interview package designed to diagnose psychiatric disorders in children and adolescents aged 5-17 years. The validity and reliability of the Turkish version of DAWBA, originally developed by Goodman et al,^11^ were established by Dursun et al.^12^ Additionally, the Wechsler Intelligence Scale for Children–Revised (WISC-R) was administered to evaluate participants’ intellectual functioning. The WISC-R is an assessment tool used to measure the intelligence level of participants. The standardization study of the WISC-R developed by Wechsler for Turkish children aged 6-16 years was conducted by Savaşır & Şahin.^13^^,^^14^

Eligible participants completed cognitive tasks (Stroop and Go/No-Go) in a soundproof, dimly lit room, seated approximately 70 cm from a computer screen. Instructions were provided both verbally and visually, and tasks commenced after confirming understanding. Task duration was approximately 20 minutes, with stimulus presentation and task control managed using E-Prime 2.0 software (Psychology Software Tools, Pittsburgh, PA). Participants were instructed to avoid consuming stimulant beverages (e.g., tea, coffee) after midnight prior to tasks.

### Stroop Task

**Figure 1.**
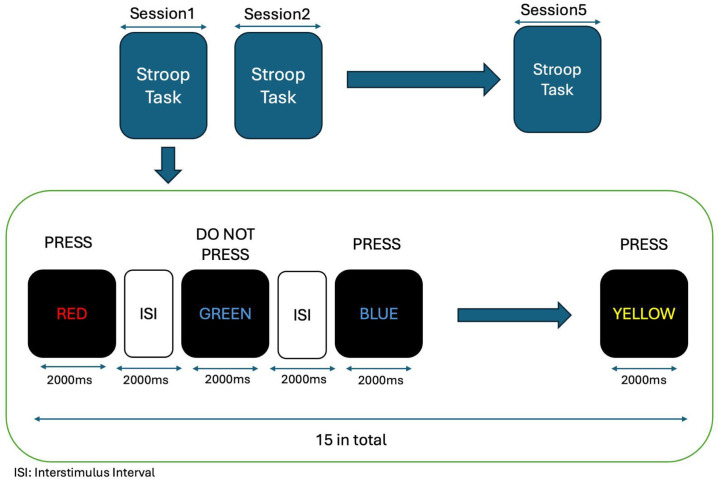
Schematic of congruent and incongruent stimulus conditions in the Stroop task.

The Stroop task assessed selective attention, cognitive flexibility, response inhibition, conflict monitoring, and information processing speed. Participants responded to color-word stimuli by pressing a button for congruent trials and withholding a response for incongruent trials.^15^ The task comprised 5 blocks of 15 stimuli each, with a 70 : 30 ratio of congruent to incongruent trials (Figure 1).^16^ Behavioral measures included reaction time, correct responses, omission errors, and commission errors. Omission errors have been associated with attentional deficits, while commission errors have been linked to impaired response inhibition.^17^

### Go/No-Go Task

**Figure 2.**
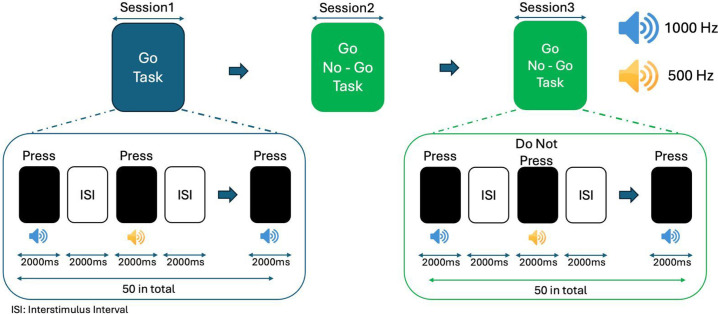
Schematic of stimulus presentation and response instructions in the Go/No-Go task.

In the Go/No-Go task, participants are required to press a button in response to a specific stimulus (Go) and to withhold their response to a different stimulus (No-Go). The Go/No-Go task enables researchers to examine response inhibition processes.^18^ In this study, the auditory Go/No-Go task was developed using MATLAB software and presented in a computerized environment. All participants completed a task in which 2 different auditory stimuli were presented at 2000-millisecond intervals via a computer. When a 1000 Hertz high-pitched tone was presented, participants were instructed to press a button (Go trial), whereas they were asked to withhold their response when a 500 Hertz low-pitched tone was presented (No-Go trial). The total duration of the auditory Go/No-Go task was approximately 12 minutes. The task comprised 3 blocks of 50 stimuli each, with a 70 : 30 ratio of Go to No-Go trials (Figure 2). Behavioral measures included reaction time, correct responses, omission errors, and commission errors.

### Event-Related Potentials

Continuous EEG was recorded using an ActiCap and ActiChamp amplifier system (Brain Products GmbH, Gilching, Germany). The ActiCap contained 32 electrode sites based on the 10/20 system. EEG data were processed offline using Brain Vision Analyzer 2 software (Brain Products GmbH, Gilching, Germany). Electrooculogram data were collected to correct for eye movements. EEG data were digitized at 1000 Hz, re-referenced to average mastoids, filtered (0.01-30 Hz), and corrected for ocular artifacts.^19^ Event-related potential analyses focused on P300 amplitude and latency^20^ at prefrontal frontal (F3, F4) and central (C3) electrodes. The P300 component is an ERP marker that reflects attentional orientation, target stimulus evaluation, and cognitive processing.^6^ In the 10-20 EEG system, the F4, F3, and C3 electrode sites are positioned over the right dorsolateral prefrontal cortex, implicated in high-level cognitive functions including attentional control, executive functioning, decision-making, and response inhibition; the left dorsolateral prefrontal cortex, associated with reasoning and working memory; and the left motor cortex, which primarily controls movements of the right side of the body, respectively.^21^^-^^23^

### Statistical Analysis

Statistical analyses were conducted using IBM SPSS Statistics, Version 24.0 (IBM Corp., Armonk, NY, USA). Depending on the distribution of the data, both parametric and non-parametric statistical methods were employed. For normally distributed variables, the 1-way ANOVA test was used, while the Kruskal–Wallis test was applied to non-normally distributed variables. Relationships between variables were examined using either Pearson or Spearman correlation analyses. A *P*-value of less than .05 was considered statistically significant, based on a 95% CI.

## Results

The ADHD group included 10 girls (43.5%) and 13 boys (56.5%) with a mean age of 12.6 ± 1.8 years. The BIF group consisted of 12 girls (57.1%) and 9 boys (42.9%) with a mean age of 12.2 ± 1.4 years. The Control group included 11 girls (50%) and 11 boys (50%) with a mean age of 12.2 ± 1.8 years. Age and sex distributions did not differ significantly across groups (*P* > .05), whereas maternal and paternal education and maternal employment differed significantly (*P* < .05; Table 1).

In the Stroop task, significant group differences were observed in reaction time (*F* = 21.799, *P* < .001) and omission errors (*χ*^2^ = 10.143, *P* = .006). Pairwise comparisons showed no differences between the ADHD and Control groups (*P* > .05), but both the ADHD vs. BIF (*t* = −6.308, *P* < .001; *U* = 130.5, *P* = .013) and the Control vs. BIF (*t* = −5.323, *P* < .001; *U* = 124, *P* = .003) comparisons were significant. In the BIF group, reaction time positively correlated with omission errors (ρ = .56, *P* = .008), and in the ADHD group, omission errors correlated positively with commission errors (ρ = .59, *P* = .004).

In the Go/No-Go task, reaction time (*F* = 6.52, *P* < .001) and commission errors (*F* = 6.79, *P* < .001) differed significantly across groups. Reaction times did not differ between the ADHD and Control groups (*P* > .05), but the BIF group was slower than both the ADHD (*t* = 3.32, *P* = .002) and Control (*t* = 3.00, *P* = .005) groups. Commission errors were higher in ADHD than both Control (*t* = 2.86, *P* = .008) and BIF (*t* = 2.92, *P* = .006) groups, whereas no difference was observed between Control and BIF groups (*P* > .05). No significant correlations were found among behavioral measures at the group level (*P* > .05).

**Figure 3.**
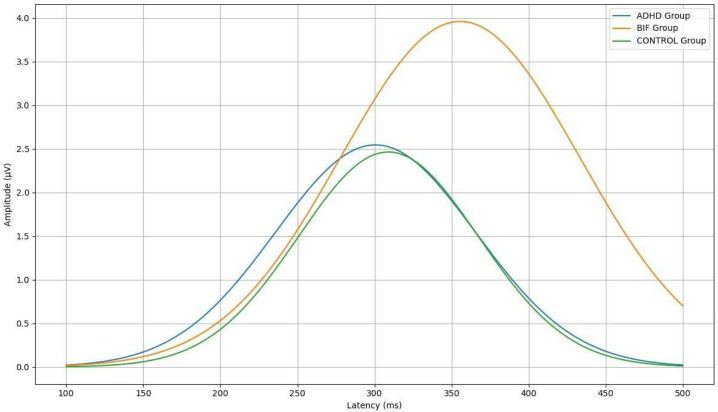
Comparison of F4 P300 latency across the groups.

Event-related potential analyses during the Stroop task revealed no significant group differences in P300 amplitude or latency at F3, F4, or C3 electrodes (*P* > .05). In the Go/No-Go task, a significant group effect was observed for F4 P300 latency (*F* = 4.171, *P* = .020), with post hoc analyses revealing prolonged latency in the BIF group relative to both ADHD (*t* = −2.552, *P* = .014) and control participants (*t* = 2.226, *P* = .032) (Figure 3); no significant differences emerged at the C3 or F3 sites (*P* > .05). Behavioral performance and ERP data are summarized in Table 2.

Correlational analyses revealed that in the Stroop task, C3 P300 latency negatively correlated with commission errors (ρ = −.58, *P* = .008) and positively correlated with correct responses (ρ = .55, *P* = .014) in the BIF group, whereas no significant correlations were observed in ADHD. In the Go/No-Go task, ADHD participants showed positive correlations between reaction time and C3 P300 amplitude (ρ = .65, *P* = .001) as well as F4 P300 amplitude (ρ = .52, *P* = .010), while in the BIF group, F3 P300 amplitude was positively associated with omission errors (ρ = .53, *P* = .013).

## Discussion

This study investigated executive function in children with ADHD, BIF, and typically developing controls using Stroop and Go/No-Go tasks in conjunction with ERP recordings. Sociodemographic characteristics were generally comparable across groups; however, lower parental education levels were observed in the BIF group, which should be considered when interpreting differences in cognitive performance and executive functioning.

Children with BIF exhibited slower reaction times and higher omission errors across tasks, reflecting limited cognitive resources, particularly in information processing, response generation, as well as attentional control, impairments in sustained attention, and reduced processing speed.^24^^,^^25^ Furthermore, the literature associates omission errors with the inability to respond to stimuli in a timely manner, suggesting that slower information processing, which prolongs reaction times, may lead to increased omission errors due to delayed responses to target stimuli.^26^ Consistent with previous research, the present study found that the BIF group demonstrated a positive correlation between reaction time and omission errors. Prolonged P300 latency at the F4 electrode further reflected delayed cognitive processing and slower stimulus evaluation, consistent with the cognitive profile of individuals with BIF, characterized by reduced information processing speed, limited attentional capacity, and constrained working memory.^27^ Furthermore, in a study by Vaney and colleagues comparing individuals with BIF to healthy controls, a significant prolongation of P300 latency was also observed in the BIF group.^10^ In the BIF group, P300 latency at the C3 electrode was negatively correlated with commission errors. Although shorter P300 latencies typically reflect faster stimulus processing and quicker response generation, individuals with BIF who have limited cognitive resources may be more prone to errors when responding rapidly due to insufficient processing and control.^28^^,^^29^ In contrast, longer P300 latencies permit more deliberate stimulus evaluation, potentially reducing error rates. Consistent with this, a positive correlation was observed between P300 latency and the number of correct responses, indicating that extended processing time supports more accurate performance in this population. F3 P300 amplitudes were significantly correlated with omission errors, reflecting limited cognitive resources in attentional processes that impair stimulus processing and response generation.^29^ Supporting the present finding, a study observed that transcranial alternating current stimulation increased P300 amplitudes and reduced omission errors.^30^

In contrast, children with ADHD demonstrated faster reaction times and increased commission errors, indicating heightened impulsivity and impaired response inhibition.^31^ Impulsivity, a core neuropsychological characteristic of ADHD, impairs an individual’s ability to regulate behavior, thereby increasing the likelihood of errors, particularly in tasks that demand inhibitory control. The literature highlights that individuals with ADHD often respond rapidly to stimuli; however, these responses are frequently inaccurate, resulting in a greater number of commission errors.^32^ Shorter P300 latency at F4 in ADHD, relative to BIF and without significant differences from controls, together with previously reported reductions in prefrontal cortex activity, indicates rapid yet potentially superficial stimulus processing and less controlled information processing, consistent with inhibitory control deficits in ADHD. Indeed, in this study, participants with ADHD not only had shorter reaction times but also made significantly more commission errors. Core neuropsychological features of ADHD, such as impulsivity and impaired response inhibition, can lead to prematurely executed responses, bypassing deeper cognitive processing and increasing the likelihood of error-prone responding.^33^^,^^34^ Correlations between P300 amplitude and reaction times at C3 (corresponding to the left motor cortex in right-hand–dominant participants) and F4 suggest that motor and frontal control processes are differentially engaged in ADHD, with motor responses relying on more limited cortical resources at C3, resulting in rapid but superficial response patterns, while frontal mechanisms at F4 are recruited selectively during certain correct responses. This variable engagement of prefrontal control mechanisms likely underlies the characteristic cognitive performance fluctuations and inconsistent cognitive control observed in individuals with ADHD.^17^^,^^35^

Several limitations should be noted. First, the relatively small sample size restricts the generalizability of the findings. In addition, differences in parental education levels between groups may have acted as potential confounding factors influencing executive functioning and cognitive performance. Furthermore, multiple electrophysiological and correlational analyses were conducted without formal correction for multiple comparisons (e.g., Bonferroni or false discovery rate adjustment), which may have increased the risk of type 1 error. Therefore, the findings should be interpreted cautiously until replicated in larger independent samples. Second, potential cognitive heterogeneity within the BIF group warrants more fine-grained subgroup analyses. Third, ERP analyses were limited to the F3, F4, and C3 sites, neglecting contributions from other cortical regions implicated in executive functioning. Finally, the restricted number and scope of behavioral tasks may have constrained the assessment of executive domains. Future studies should recruit larger and more homogeneous samples, employ a broader battery of tasks, and include multiregional neural analyses to better delineate the neural substrates of executive dysfunction in ADHD and BIF.

This study investigated executive functions in individuals with ADHD, BIF, and healthy controls using behavioral tasks and ERPs. Groups were similar in basic demographics, strengthening the study’s validity. Individuals with BIF demonstrated slower reaction times, fewer correct responses, and more omission errors, consistent with reduced information processing speed and impaired attentional continuity. At the neural level, prolonged P300 latency in the right dorsolateral prefrontal cortex (F4 region) further indicated cognitive slowing. In contrast, participants with ADHD exhibited faster reaction times but increased commission errors, reflecting impulsivity and deficits in response inhibition. Their shorter P300 latency suggested rapid but less controlled cognitive processing. Together, these findings may reflect partially distinct behavioral and neurophysiological patterns of executive dysfunction in ADHD and BIF, particularly in domains of attention control and inhibitory processes. Event-related potential measures may provide preliminary neurophysiological insights that could contribute to understanding executive dysfunction in these populations; however, larger studies are needed before drawing definitive diagnostic implications or clinical applications.

## Figures and Tables

**Table 1.** Comparison of Sociodemographic Characteristics Across Groups

Variables	ADHD Group	BIF Group	Control Group	*P*
	(n = 23)	(n = 21)	(n = 21)	
Gender, n (%)				.664
Male	13 (56.5)	12 (57.1)	11 (50)	
Female	10 (43.5)	9 (42.9)	11 (50)	
Mother’s age (years) (mean ± SD)	40.8 ± 3.7	39.7 ± 3.8	40.9 ± 5.3	.602
Father’s age (years) (mean ± SD)	42.7 ± 4.6	42.5 ± 5.7	43.6 ± 5.4	.772
Education level of mother, n (%)				.024*
Primary and secondary school	6 (26.1)	11 (52.4)	8 (36.4)	
High school	8 (34.8)	10 (47.6)	6 (27.3)	
University	9 (39.1)	0 (0)	8 (36.4)	
Education level of father, n (%)				.002**
Primary and secondary school	3 (13)	9 (42.9)	6 (27.3)	
High school	7 (30.4)	11 (52.4)	4 (18.2)	
University	13 (56.5)	1 (4.8)	12 (54.5)	
Family type, n (%)				.275
Nuclear	22 (95.7)	17 (81)	20 (90.9)	
Extended	1 (4.3)	4 (19)	2 (9.1)	
Place of residence, n (%)				.325
Urban	22 (95.7)	19 (90.5)	22 (100)	
Rural	1 (4.3)	2 (9.5)	0 (0)	
Family income level, n (%)				.121
Less than minimum wage	8 (34.8)	10 (47.6)	4 (18.2)	
The minimum wage and above	15 (65.2)	11 (52.4)	18 (81.8)	

ADHD, attention-deficit/hyperactivity disorder; BIF, borderline intellectual functioning. Bold values indicate statistically significant results.

*Used for *P*-values <.05.

**Used for *P*-values <.01.

**Table 2.** Behavioral and P300 Comparisons Across Stroop and Go/No-Go Tasks in ADHD, BIF, and Control Groups

Variables	ADHD Group	BIF Group	Control Group	*P*
	(n = 23)	(n = 21)	(n = 21)	
Correct responses	72.1 ± 5.6	68.9 ± 9.5	73.6 ± 1.3	.051
Average reaction time	745.6 ± 138.2	1007.5 ± 130.9	776.9 ± 151.8	<.001***
Omission error	1.2 ± 3.5	3.7 ± 7.4	0.4 ± 0.5	<.01**
Commission error	1.7 ± 2.4	2.4 ± 3.1	1 ± 1.2	.152
F3 (mean ± SD)				
Amplitude (µV)	2.5 ± 1.4	5.3 ± 7.7	4.1 ± 5.9	.294
Latency (milliseconds)	637.3 ± 201.5	672.7 ± 242.4	642.6 ± 193.4	.859
F4 (mean ± SD)				
Amplitude (µV)	4 ± 3.6	3.2 ± 2.8	4.3 ± 4.3	.649
Latency (milliseconds)	648.6 ± 254.1	659.8 ± 242.2	654.2 ± 233.4	.99
C3 (mean ± SD)				
Amplitude (µV)	4.9 ± 1.7	8.4 ± 1.5	5.9 ± 2.9	.413
Latency (milliseconds)	662.1 ± 178.7	710.5 ± 163.7	762.6 ± 100.9	.108
Go/No-Go task (mean ± SD)				
Correct responses	85.8 ± 15.6	90.9 ± 14.2	94.6 ± 3.5	.058
Average reaction time	503.9 ± 179.1	665.9 ± 139.4	535.1 ± 145.8	.003**
Omission error	2.6 ± 4.4	2.3 ± 7.1	0.7 ± 1.0	.483
Commission error	9.3 ± 7.1	4.3 ± 3.8	4.7 ± 3.1	.002**
F3 (mean ± SD)				
Amplitude (µV)	3.4 ± 3.8	3.5 ± 3.3	4.9 ± 3.9	.283
Latency (milliseconds)	322.9 ± 64.8	326 ± 65.9	297.3 ± 69.7	.401
F4 (mean ± SD)				
Amplitude (µV)	6.8 ± 1.1	4.5 ± 3.5	3.7 ± 3.2	.647
Latency (milliseconds)	300.6 ± 64.9	355.5 ± 77.6	309 ± 58.4	.020*
C3 (mean ± SD)				
Amplitude (µV)	5.4 ± 4.9	5.3 ± 3.9	6.2 ± 3.2	.44
Latency (milliseconds)	335.3 ± 88.8	336.8 ± 98.7	301.5 ± 63.4	.353

ADHD, attention-deficit/hyperactivity disorder; BIF, borderline intellectual functioning. Bold values indicate statistically significant results.

*Used for *P*-values < .05.

**Used for *P*-values < .01.

***Used for *P*-values < .001.

## Data Availability

he data that support the findings of this study are available on request from the corresponding author.
